# Post-marketing surveillance of encorafenib in combination with binimetinib in Japanese patients with *BRAF*-mutant melanoma

**DOI:** 10.1007/s10147-025-02693-6

**Published:** 2025-02-07

**Authors:** Naoya Yamazaki, Hidenori Sakata, Osamu Iida, Teruaki Katayama, Hisashi Uhara

**Affiliations:** 1 Department of Dermatologic Oncology, National Cancer Center Hospital, Tokyo, Japan; 2 Department of Pharmacovigilance, Ono Pharmaceutical Co., Ltd, Osaka, Japan; 3 Department of Oncology Medical Affairs, Ono Pharmaceutical Co., Ltd, Osaka, Japan; 4 Department of Dermatology, Sapporo Medical University, South 1, West 16, Chuo-Ku, Sapporo, Hokkaido 060-8556 Japan

**Keywords:** Encorafenib, Binimetinib, Post-marketing surveillance, Malignant Melanoma, Japan, *BRAF* mutation

## Abstract

**Background:**

A BRAF inhibitor, encorafenib, combined with a MEK inhibitor, binimetinib, was approved in Japan in early 2019 for the treatment of *BRAF* V600-mutant, unresectable malignant melanoma based on results of the global phase III trial, COLUMBUS, conducted in various countries including Japan. This post-marketing surveillance (PMS) assessed the combination in real-world clinical practice in Japan.

**Methods:**

We performed a prospective, multicentre, 12-month PMS of the safety and effectiveness of encorafenib plus binimetinib for radically unresectable, *BRAF*-mutant malignant melanoma in Japan.

**Results:**

Among 174 survey forms collected from 85 centres between February 2019 and August 2020, 172 were included for safety and effectiveness analysis. Patients (male [52.3%], median age 62.0 years) had Eastern Cooperative Oncology Group Performance Status 0 or 1 (91.8%) and comorbidities (55.2%). Respective encorafenib and binimetinib median dosages were 450 mg/day and 90 mg/day; median treatment duration, 24.1 and 24.2 weeks, and discontinuation, 71.5% for each, primarily for disease progression (56.9%) and adverse drug reactions (ADRs, 38.2%). Safety assessment ADRs occurred in 99 patients (57.6%), including eye disorders (40.7%), hepatic dysfunction (20.3%), rhabdomyolysis (4.7%), haemorrhage (2.3%), palmar-plantar erythrodysaesthesia syndrome (1.7%), and hypertension (1.7%); 19.8% were grade ≥ 3, none were grade 5, most resolved with/without treatment modification. At 12 months, the objective response rate was 48.8% (95% CI 41.2, 56.6; complete [19.2%], partial [29.7%]), overall survival was 40.1%.

**Conclusion:**

The safety and effectiveness of encorafenib plus binimetinib in Japanese patients with *BRAF*-mutant malignant melanoma were similar to data reported in COLUMBUS; no new safety concerns were identified.

**Supplementary Information:**

The online version contains supplementary material available at 10.1007/s10147-025-02693-6.

## Introduction

Cutaneous melanoma is a malignancy of melanocytes for which the incidence varies globally, and in some regions is increasing [1]. In Japan, where malignant melanoma is considered a rare type of cancer, the reported incidence is 1.75 per 100,000 individuals [2]. Among the genetically distinct clinical subtypes, acral lentiginous melanoma was shown to be the most common (40.4%), followed by superficial spreading melanoma (20.5%), nodular melanoma (10.0%), mucosal melanoma (9.5%) and lentigo maligna melanoma (8.1%) [3]. With disease progression, the survival prognosis is poor; the 5-year survival rate for stage III disease is an estimated 41.7–75.0%, 17.7% for stage IV disease, and acral lentiginous melanoma is associated with the worst outcomes in patients with stage IIIa disease [3].

Mutations in the Raf-MEK-ERK mitogen-activated protein kinase (MAPK) cell proliferation signalling pathway, including *BRAF*, *NRAS* and *KIT* mutations, are frequently detected in patients with malignant melanoma; the *BRAF* mutation rate in the Japanese population is about 30% [4]. Combination treatment with BRAF (vemurafenib, dabrafenib or encorafenib) and downstream MEK (trametinib or binimetinib) inhibitors is recommended for patients with advanced *BRAF*-mutant melanoma [5–7].

Based on the results of the global, phase III COLUMBUS trial [8, 9], the BRAF/MEK inhibitor combination, encorafenib plus binimetinib, was approved in Japan in early 2019 for the treatment of *BRAF*-mutant, at the V600 locus (*BRAF* V600), unresectable malignant melanoma [10]. In COLUMBUS, median overall survival (OS) for patients who received the approved dosages of encorafenib (450 mg once daily) plus binimetinib (45 mg twice daily) was significantly longer than for those who received vemurafenib 960 mg twice daily: 33.6 months vs 16.9 months (hazard ratio [HR] 0.61; 95% confidence interval [CI], 0.47–0.79; p < 0.0001) [9].

As only three Japanese patients were included at the approval dose in COLUMBUS [9], real-world clinical data collection was deemed necessary and, in accordance with the Ministry of Health, Labor and Welfare of Japan, this post-marketing surveillance (PMS) study was conducted as part of the approval condition for encorafenib plus binimetinib. In the encorafenib plus binimetinib group in the COLUMBUS trial, safety specifications (SS) were identified, including cutaneous malignant tumours, palmar-plantar erythrodysaesthesia syndrome, eye disorders, cardiac dysfunction, hypertension, rhabdomyolysis, hepatic dysfunction and haemorrhage [8, 11]. The aim of this study was to investigate the safety and effectiveness of encorafenib plus binimetinib to understand the frequency and characteristics of these SS in real-world clinical practice in Japan. In particular, eye disorders are a class effect of MEK inhibitors [12–14], hence detailed analyses of these adverse events (AEs) were conducted, including time to onset and recovery or improvement, and treatment status.

## Patients and methods

### Study design

We performed a prospective, multicentre, observational PMS study of patients with radically unresectable, *BRAF*-mutant malignant melanoma in Japan who received at least one dose of encorafenib plus binimetinib (Clinical Research Submission and Disclosure System registration number: jRCT2011210010). The study was conducted in accordance with the ministerial ordinance on Standards for Conducting Surveys and Studies after the Manufacture and Sale of Drugs in Japan, which determines that ethical committee approval and informed consent from patients was not required, and hence for this PMS, was not obtained. Each medical centre which agreed to participate in this survey entered into a written contract with the study sponsor. The survey was conducted via a central registration system and the registration period for collecting survey forms was from February 26, 2019 through to August 31, 2020. The observation period for each patient was 12 months from the start of encorafenib plus binimetinib combination therapy.

### Patients and treatment

Patient data collected using case report forms (CRFs) included demographic and clinical characteristics (Eastern Cooperative Oncology Group Performance Status [ECOG PS]), comorbidities, disease type and stage, number of previous treatments, and recent treatment. The daily dose of encorafenib (approved dosage 450 mg once daily) plus binimetinib (45 mg twice daily), treatment duration, and whether treatment was discontinued (with reasons), were recorded.

### Assessments

The primary outcome was the incidence of safety assessment items that occurred during the 12-month period from the start of encorafenib plus binimetinib combination therapy, or until 30 days after the last use of either drug if a patient discontinued the use of both agents, whichever was later. The safety assessment items under the category of safety specifications were defined based on SS prespecified in the risk management plan for encorafenib plus binimetinib: cutaneous malignant tumours; palmar-plantar erythrodysaesthesia syndrome; eye disorders; cardiac dysfunction; hypertension; rhabdomyolysis; hepatic dysfunction and haemorrhage. Adverse drug reactions (ADRs) associated with these safety assessment items in patients with unresectable *BRAF*-mutant malignant melanoma were collected. The definition of rhabdomyolysis in this study also included high creatine phosphokinase (CPK) values and muscle pain; for a full list of definitions of the ADRs collected for the safety assessment see Supplementary Table 1.

The time to occurrence and outcomes of AEs for each Preferred Term (PT) related to eye disorders were analysed. Additionally, for eye disorders, the outcomes by severity (grade 1 to 5) and by treatment disposition were assessed, and complications and incidence of eye disorders were evaluated. Other medical histories and AEs apart from malignant melanoma were coded using MedDRA/J version 25.1. AEs were evaluated based on the severity grade, according to the National Cancer Institute’s Common Terminology Criteria for Adverse Events (CTCAE).

The best tumour response, according to Response Evaluation Criteria in Solid Tumors (RECIST) version 1.1, and patient outcomes were evaluated during the first 12 months of initiating encorafenib plus binimetinib treatment, or until discontinuation of the two-drug combination, whichever came first.

### Statistical analysis

Based on the incidence of ADRs associated with the safety assessment items in the encorafenib plus binimetinib group in the COLUMBUS trial [8], the number of cases planned for this PMS was 150 to permit the detection of an ADRs incidence rate of 2.1% with a probability of 95.9%. Statistical analyses were performed using the Statistical Analysis System (SAS) Version 9.4 (SAS Institute Inc., Cary, NC, USA).

## Results

### Patient characteristics

**Fig. 1:**
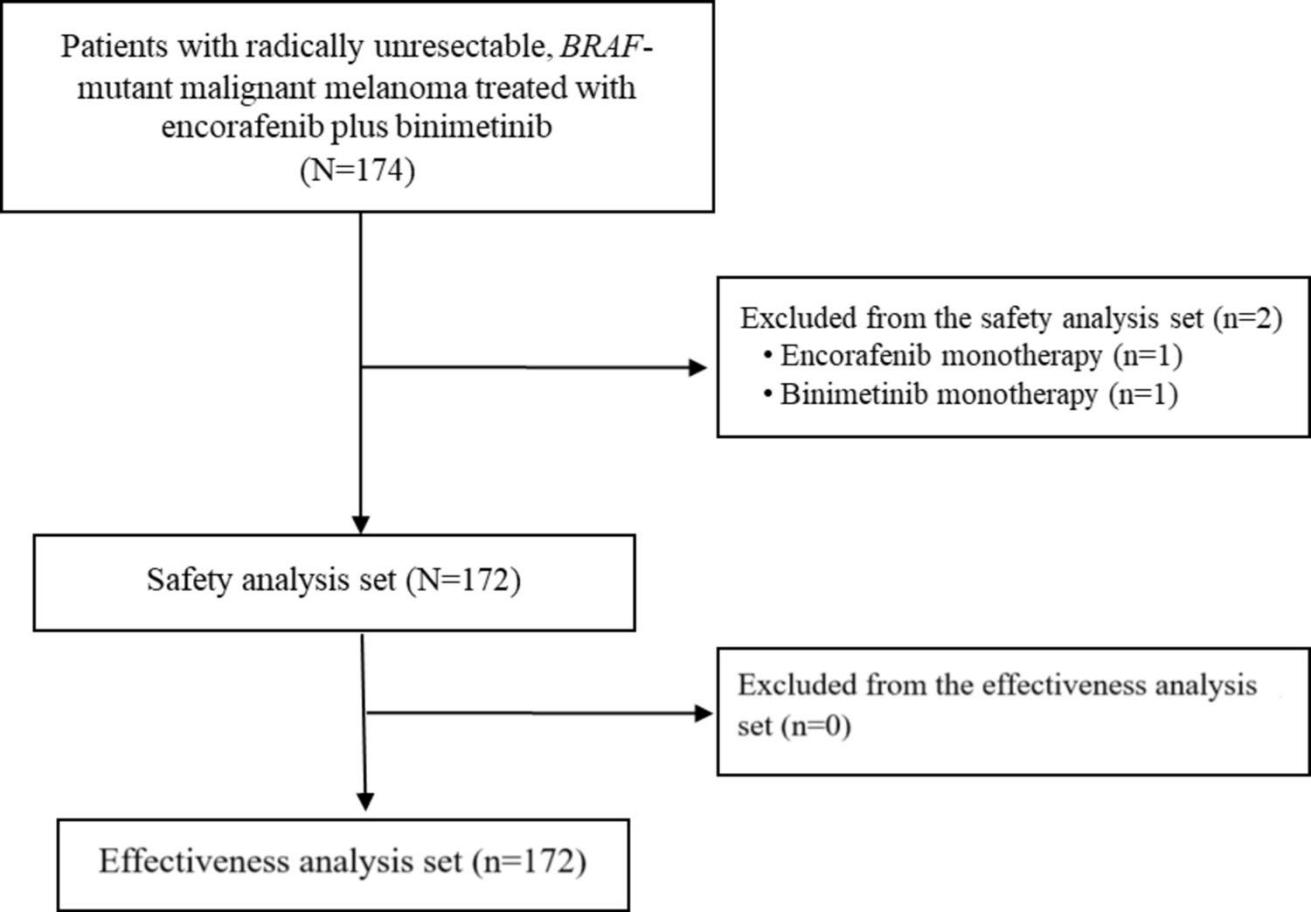
Flow chart of the study survey population

Overall, 174 CRFs were collected from 85 centres during the 18-month registration period. Among these, two were excluded from the safety analysis set, one case each of encorafenib monotherapy and binimetinib monotherapy (Fig. 1). In total, 172 cases were included in the safety and effectiveness analysis sets.

Patients’ characteristics are summarized in Table 1. Almost half were male (52.3%), the median age was 62 (range: 25–87) years, and 18.0% were aged ≥ 75 years. Most patients (91.8%) had an ECOG PS of 0 or 1 and over half (55.2%) had comorbidities; by affected organ, these included the heart (9.3%), liver (9.9%), eye (8.7%), kidney (2.9%), or other (46.5%). By disease stage classification, 24 patients were stage III (14.0%) and 145 stage IV (84.3%); 3 (1.8%) were other.

**Table 1:** Patient characteristics, safety analysis set (*N* = 172)

Characteristics, *n (%)*^*a*^	Total (N = 172)
Sex	
Male	90 (52.3)
Female	82 (47.7)
Age, years	
Median (range)	62.0 (25–87)
< 75	141 (82.0)
≥ 75	31 (18.0)
ECOG performance status	
0	121 (70.3)
1	37 (21.5)
2	8 (4.7)
3	5 (2.9)
4	1 (0.6)
Comorbidities	
Yes	95 (55.2)
Heart	16 (9.3)
Liver	17 (9.9)
Eyes	15 (8.7)
Kidney	5 (2.9)
Other	80 (46.5)
Malignant melanoma status at start of treatment^b^	
Initial onset	52 (30.2)
Recurrence	114 (66.3)
Unknown	6 (3.5)
Metastasis, site^c^	172 (100.0)
Brain	41 (23.8)
Lung	73 (42.4)
Visceral organs	71 (41.3)
Skin	53 (30.8)
Lymph nodes	108 (62.8)
Muscles and soft tissue	23 (13.4)
Other	46 (26.7)
Staging	
III	24 (14.0)
IV	145 (84.3)
Other	3 (1.7)
Malignant melanoma treatment history	
Surgery	146 (84.9)
Radiotherapy	42 (24.4)
Adjuvant chemotherapy	59 (34.3)
Pharmacotherapy excluding adjuvant chemotherapy	114 (66.3)
Previous lines of treatment	
None	58 (33.7)
First-line	47 (27.3)
Up to second-line	39 (22.7)
Third-line or more	26 (15.1)
Unknown	2 (1.2)
Most recent treatment	
Anti-PD-1 antibody	42 (36.8)
Nivolumab	31 (27.2)
Pembrolizumab	11 (9.6)
Ipilimumab	1 (0.9)
Nivolumab + ipilimumab	24 (21.1)
Dabrafenib + trametinib	42 (36.8)
Dacarbazine (DTIC)	2 (1.8)
Others	4 (3.5)
Time from the end of the most recent treatment to the start of encorafenib or binimetinib treatment^d^, days	
Median (min, max)	23.0 (1, 324)
Use of immune checkpoint inhibitors	45 (39.5)

^a^Unless otherwise reported

^b^Encorafenib in combination with binimetinib

^c^Including multiple sites

^d^Whichever was earliest

*max* maximum, *min* minimum

The number of previous treatments was 0 for 58 patients (33.7%), 1 for 47 patients (27.3%), 2 for 39 (22.7%), 3 for 26 (15.1%), and unknown for 2 patients (1.2%). The most recent treatments in the 114 previously treated patients included dabrafenib plus trametinib (36.8%), nivolumab (27.2%), nivolumab plus ipilimumab (21.1%), and pembrolizumab (9.6%).

### Treatment status

The median (range) dosages of encorafenib and binimetinib were 450 (124.4–450.0) mg/day and 90 (19.8–90.0) mg/day, respectively (Table 2); respective median treatment durations were 24.1 (0.1–52.1) weeks and 24.2 (0.1–52.1) weeks. The discontinuation rates of encorafenib or binimetinib were 71.5% (123/172) each and, for both treatments, the main reasons for discontinuation were disease progression (including death), 56.9% (70/123), and AEs, 38.2% (47/123) (Table 2).

**Table 2:** Treatment status: dosage, duration, discontinuation (*N* = 172)

Encorafenib		Binimetinib	
Dose, mg/day^a^	*n (%)*	Dose, mg/day^a^	*n (%)*
Median (min–max)	450 (124.4–450.0)	Median (min–max)	90.0 (19.8–90.0)
< 200	2 (1.2)	< 30	3 (1.7)
200 to < 300	16 (9.3)	30 to < 60	16 (9.3)
300 to < 450	54 (31.4)	60 to < 90	53 (30.8)
450 ≤	99 (57.6)	90 ≤	99 (57.6)
Unknown	1 (0.6)	Unknown	1 (0.6)

Treatment duration, weeks		Treatment duration, weeks	
Median (min–max)	24.1 (0.1–52.1)	Median (min–max)	24.2 (0.1–52.1)
< 12	49 (28.5)	< 12	49 (28.5)
12 – < 24	37 (21.5)	12 – < 24	37 (21.5)
24 – < 36	25 (14.5)	24 – < 36	25 (14.5)
36 – < 48	12 (7.0)	36 – < 48	12 (7.0)
48 ≤	49 (28.5)	48 ≤	49 (28.5)

Treatment discontinuation		
	Encorafenib	Binimetinib
Discontinued treatment, *n (%)*	123 (71.5)	123 (71.5)
Reason for discontinuation^b^, *n (%)*		
Primary disease progression (including death)	70 (56.9)	70 (56.9)
Occurrence of AEs	47 (38.2)	47 (38.2)
Other	8 (6.5)	8 (6.5)
Transfer to another hospital	3 (2.4)	3 (2.4)

^a^Total dosage/duration of treatment (excluding treatment interruption)

^b^Multiple choices permitted

*AE* adverse event, *max* maximum, *min* minimum

### Safety

In the safety analysis population (N = 172), the frequency of ADRs associated with SS was 57.6%; 19.8% were assessed as grade ≥ 3 and none as grade 5 (Table 3). Eye disorders (40.7%) were the most common ADR followed by hepatic dysfunction (20.3%), rhabdomyolysis (4.7%), haemorrhage (2.3%), palmar-plantar erythrodysaesthesia syndrome (1.7%), hypertension (1.7%), and cardiac dysfunction (1.2%); there were no reports of cutaneous malignant tumours (Table 3).

**Table 3:** Incidence of ADRs ≥ 1.5% by safety specification and PT in patients treated with encorafenib 450 mg once daily in combination with binimetinib 45 mg twice daily (*N* = 172)

ADR, *n (%)*	Any grade	Grade ≥ 3
All	99 (57.6)	34 (19.8)
Eye disorders	70 (40.7)	13 (7.6)
Serous retinal detachment	31 (18.0)	7 (4.1)
Vision blurred	7 (4.1)	1 (0.6)
Eye disorder	6 (3.5)	
Serous retinopathy	5 (2.9)	1 (0.6)
Retinal detachment	4 (2.3)	
Uveitis	4 (2.3)	2 (1.2)
Visual impairment	4 (2.3)	1 (0.6)
Visual acuity reduced	3 (1.7)	1 (0.6)
Hepatic dysfunction	35 (20.3)	15 (8.7)
Hepatic function normal	20 (11.6)	8 (4.7)
Liver disorder	8 (4.7)	4 (2.3)
Alanine aminotransferase increased	3 (1.7)	
Rhabdomyolysis	8 (4.7)	3 (1.7)
Blood creatine phosphokinase increased	4 (2.3)	
Rhabdomyolysis	3 (1.7)	2 (1.2)
Haemorrhage	4 (2.3)	3 (1.7)
Hypertension	3 (1.7)	2 (1.2)
Hypertension	3 (1.7)	2 (1.2)
Palmar-plantar erythrodysaesthesia syndrome	3 (1.7)	0 (0.0)
Palmar-plantar erythrodysaesthesia syndrome	3 (1.7)	
Cardiac dysfunction	2 (1.2)	0 (0.0)
Malignant skin tumours	0 (0.0)	0 (0.0)

The types of ADRs are reported using the MedDRA/J, Version 25.1

If multiple episodes of the same ADR occurred in a patient, the ADR was included in the highest-grade category and counted as one event only

Cases of AEs of unknown grade were excluded from the Any Grade column of the table

AEs for which a causal relationship to either encorafenib or binimetinib could not be ruled out were handled as ADRs

Proportion (%) was calculated with the number of patients included in the Safety Analysis Set as the denominator

*ADR* Adverse drug reaction, *AE* Adverse event, *PT* Preferred term

**Fig. 2:**
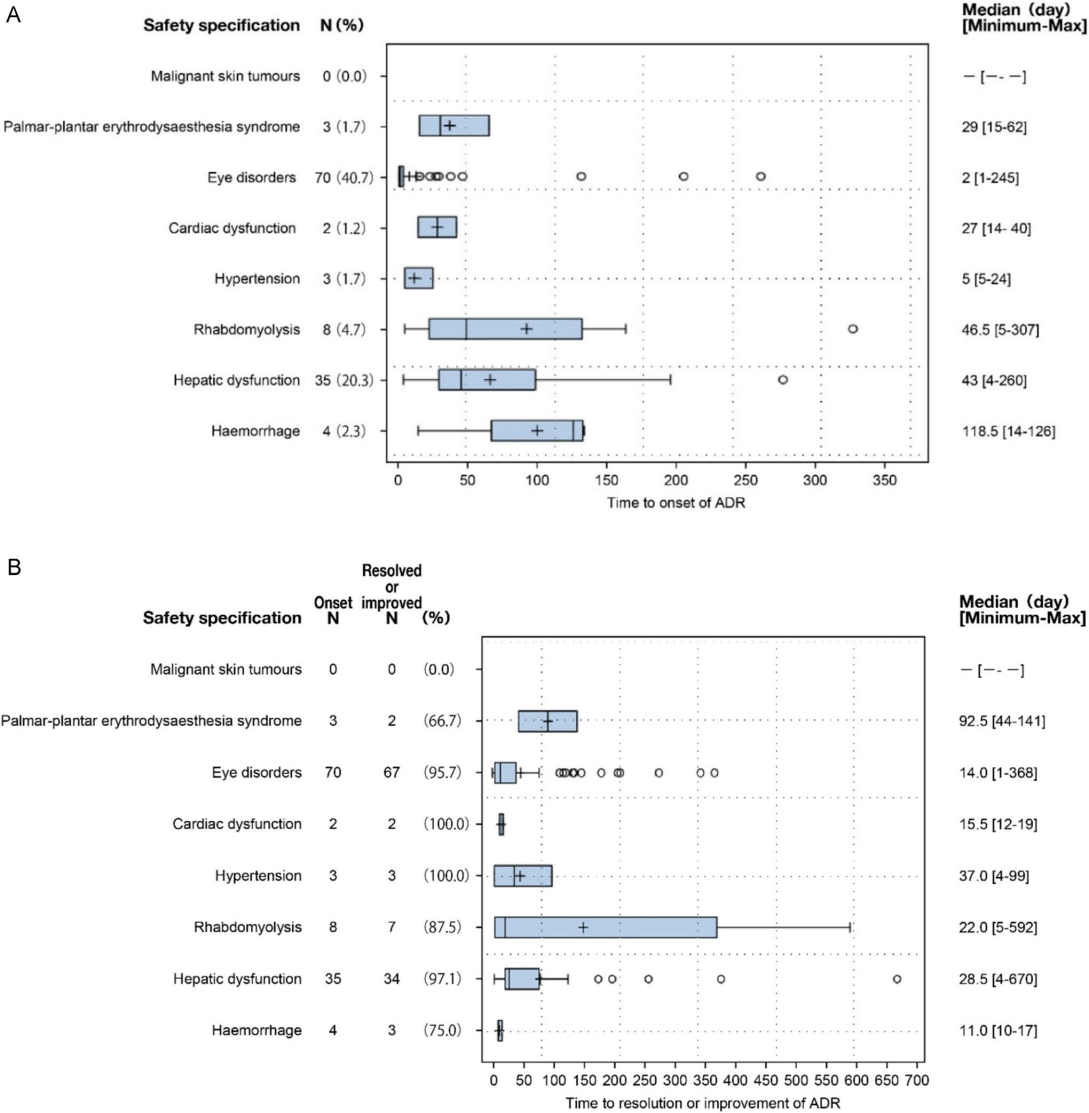
Time to onset of ADRs classified as safety specifications of encorafenib plus binimetinib treatment (Fig. 2**A**) and time to resolution or improvement of ADRs classified as safety specifications of encorafenib plus binimetinib treatment (Fig. 2**B**). Time (days) to onset of ADRs was from the start date of encorafenib or binimetinib treatment, whichever was earliest. Adverse events for which a causal relationship to either encorafenib or binimetinib could not be ruled out were considered ADRs. *ADR* adverse drug reaction

The median time to occurrence of ADRs was < 50 days, with the exception of haemorrhage, which occurred at 118.5 days from the start of encorafenib plus binimetinib combination therapy (Fig. 2A). The proportion of patients who recovered or improved following an ADR was 66.7–100.0% (Fig. 2B). For eye disorders and hepatic dysfunction, which occurred at the highest frequency, almost all patients recovered or improved, 95.7% (67/70) and 97.1% (34/35), respectively. The median time to recovery post ADR was within 40 days, with the exception of post palmar-plantar erythrodysaesthesia syndrome, for which the median recovery time was 92.5 (44–141) days (Fig. 2B).

The individual incidences of eye disorders were 18.0% for serous retinal detachment (SRD), blurred vision (4.1%), eye disorders (3.5%), and serous retinopathy (2.9%) (Table 3). The median time to occurrence of each eye disorder, excluding uveitis which had a median of 76 days, was 1.5–4 days (Supplementary Fig. 1); recovery or improvement rates for each ranged from 71.4–100% (Supplementary Table 2). The median time to recovery or improvement from eye disorders, excluding uveitis which had a median of 148 days, was 4–15 days (Supplementary Fig. 2). The recovery or improvement rates for eye disorders by grade 1, 2, 3, and 4 were 97.3%, 95.0%, 91.7%, and 100%, respectively, and time to resolution or improvement was 10–15 days for grades 1–3, and 43 days for grade 4 (Supplementary Table 3). Treatment status following eye disorders included discontinuation for 19 patients, temporary cessation for 35, dose reduction for 9, and continuation for 33 patients; most ADRs recovered or improved following each action, with resolution or improvement rates of 97.0%, 100.0%, 97.1%, and 84.2%, respectively (Supplementary Table 4). A few unresolved cases of SRD, blurred vision, and uveitis were observed; however, most resolved or were improved (Supplementary Table 4).

Stratified analysis of the incidence of eye disorders showed that SRD, with or without complications, occurred in 6.7% (1/15) and 19.2% (30/156) of patients, blurred vision in 13.3% (2/15) and 3.2% (5/156), uveitis in 6.7% (1/15) and 1.9% (3/156), and visual acuity reduced in 6.7% (1/15) and 1.3% (2/156), respectively (Supplementary Table 5). The ADRs that occurred without complications were eye disorders, serous retinopathy, retinal detachment and visual impairment.

### Tumour response

In the effectiveness analysis set (N = 172), during the first 12 months of initiating encorafenib plus binimetinib combination therapy, or until treatment discontinuation, whichever came first, the objective response rate (ORR) according to RECIST version 1.1 was 48.8% (95% CI 41.2, 56.6); complete and partial responses were 19.2% and 29.7%, respectively (Table 4). OS 12 months after starting treatment was 40.1% (Table 4).

**Table 4:** Best overall responses in patients with effectiveness assessments (*N* = 172)

	Physician-reported responses by RECIST version 1.1	95% (CI)^a^
Best overall tumour response, *n (%)*		
Complete response	33 (19.2)	
Partial response	51 (29.7)	
Stable disease	39 (22.7)	
Progressive disease	24 (14.0)	
Not evaluable	25 (14.5)	
Objective response rate^b^	84 (48.8)	(41.2, 56.6)
12-month overall survival^c^, *n (%)*		
Survival	69 (40.1)	
Death	54 (31.4)	
Outcome unknown	49 (28.5)	

^a^Calculated by the exact method

^b^Defined as the proportion of patients with complete response or partial response

^c^12 months after the start of encorafenib plus binimetinib treatment, or at the time of the last use of either drug, whichever came later

*CI* Confidence interval

## Discussion

In this prospective PMS study of encorafenib plus binimetinib combination therapy in Japanese patients with *BRAF*-mutant unresectable malignant melanoma, no new safety concerns were reported when compared with results from the global phase III, COLUMBUS trial [8]. This real-world study had a shorter observation period (12.0 months) compared to the COLUMBUS trial (33.6 months) and included some patient groups not represented in the trial’s entry criteria, making direct comparison of the results difficult.

Most patient characteristics were similar between those in this study and in the COLUMBUS trial [8]. However, there were some notable differences. Patients with ECOG PS ≥ 2 (8.1%) were included in this study but were excluded from the COLUMBUS trial. Additionally, only patients who were untreated or had received immunotherapy as first-line treatment were included in COLUMBUS. In contrast, 37.8% of patients in our study received encorafenib plus binimetinib combination therapy as third-line treatment or later. Such differences in patient backgrounds were considered to potentially impact the assessment of effectiveness.

In this study in Japanese patients with malignant melanoma, the only ADRs in SS that tended to have numerically higher frequencies compared with the COLUMBUS trial were eye disorders and hepatic dysfunction of any grade, and by grade ≥ 3, eye disorders and haemorrhage. However, with the exception of grade ≥ 3 eye disorders, there were no differences in the incidences of each ADR compared with the COLUMBUS trial [8]. Although there were differences for other ADRs, the incidence rates were generally lower in the current PMS study compared with the COLUMBUS trial [8]. However, it is important to note that the ADRs in SS defined in this study included multiple PTs. For example, rhabdomyolysis included PTs corresponding to the MedDRA Standardised MedDRA Queries (SMQ) rhabdomyolysis/myopathy (broad), including rhabdomyolysis, elevated CPK levels, and muscle pain; therefore, caution is necessary in interpreting the results. The incidence of PTs classified as rhabdomyolysis was 1.7% in this study and 1.0% in COLUMBUS [8]. Additionally, a PMS of dabrafenib plus trametinib combination therapy in Japanese patients with malignant melanoma reported an incidence of 2.2% [15], similar to that reported with encorafenib plus binimetinib combination therapy. Nevertheless, despite the differences between this study and the COLUMBUS trial in the severity and incidence of ADRs, no new safety concerns were identified, suggesting that usual caution is required in the use of encorafenib plus binimetinib combination therapy in patients with malignant melanoma.

Although the incidence of eye disorders was greater in this PMS than in COLUMBUS, the phase III trial excluded patients with a history or symptoms of retinal vein occlusion (RVO) or risk factors for RVO (e.g., uncontrolled glaucoma or high intraocular pressure, hyperviscosity or hypercoagulable syndromes), and patients with a history of treatment with BRAF or MEK inhibitors, which were SS in the COLUMBUS trial [8]. This PMS did not exclude such patients, and the higher incidence of grade ≥ 3 eye disorders in this survey than in the COLUMBUS trial may be due to differences in patient characteristics at baseline; however, the exact cause is not clear. In our study, stratified analysis of the incidence of complications and AEs associated with MEK inhibitors did not show a higher rate in patients with complications. Although there were numerical differences in the incidence of AEs by item, the analysis was based on a small number of cases, and no definitive conclusions could be drawn.

Eye disorders are considered a class effect of MEK inhibitors [12–14] and were reported as AEs with dabrafenib plus trametinib combination therapy in international phase III trials (COMBI-d and COMBI-v) [16, 17] and in a PMS of Japanese patients with malignant melanoma [15]. The mechanism of the ocular effects of MEK inhibitors is not fully understood, but a rabbit model of RVO has been developed to investigate this further [18].

Gene expression profiling of retinal tissue showed changes in genes involved in oxidative stress induction, disruption of the blood-retinal barrier, and inflammation due to inhibition of the MEK signalling pathway. Preclinical studies suggest that MEK inhibition causes acute retinal pigment epithelium (RPE) toxicity, leading to excessive permeability of the RPE and disruption of the blood-retinal barrier [19].

Several reports have described the timing and outcomes of ophthalmic complications associated with MEK inhibitors, including a report of eye disorders with binimetinib in patients with malignant melanoma, of whom 40%–65% showed ocular conditions similar to central serous chorioretinopathy [20]. Most retinopathy events were grade 1–2 and many occurred within 4 weeks of starting treatment. Optical coherence tomography (OCT) revealed a dose-dependent thickening and increased volume of the central retina after treatment initiation. Subsequently, despite treatment continuation, there was a marked decrease in the thickening or increased volume of the central retina, which was associated with symptom resolution [20]. Another report presented a case of rapid changes in SRD during treatment with encorafenib plus binimetinib combination therapy in patients with melanoma; OCT revealed a shallow tent-shaped SRD in the fovea that resolved within 2 h [21].

Ocular complications with MEK inhibitor treatment often involve retinopathy that manifests within hours to weeks of treatment initiation, with recovery upon drug interruption, dose reduction or discontinuation in most cases [22, 23]. The median onset of AEs associated with MEK retinopathy in this study was 1.5–4.0 days and, consistent with previous reports, prompt recovery or improvement was achieved with either treatment discontinuation, interruption or dose reduction. These results are consistent with previous reports and suggest that, in the event of eye disorders, it is necessary to adjust the treatment by discontinuation, interruption, or reducing the dosage of the medication.

Uveitis has been reported as a class effect of BRAF inhibitors [24], with the onset ranging from several weeks to months after treatment initiation [25, 26]. With dabrafenib treatment, uveitis occurred at 10 weeks post-treatment initiation [24]; similarly, the median onset of uveitis in our study was 76 days. The frequency of any-grade uveitis with encorafenib plus binimetinib combination therapy was 2.3% in our study, consistent with data reported in a PMS study in Japanese patients treated with dabrafenib plus trametinib combination therapy (1.8%) [15]. Additionally, the time to recovery appeared to be longer for uveitis compared with SRD and macular oedema associated with MEK retinopathy.

Although the outcomes of eye disorders varied by severity, recovery or improvement rates were similar across severity grades, with a median time to recovery of 10–15 days for grades 1–3. Notably, for one grade 4 ADR, the recovery period was within the range of the minimum and maximum values for grades 1–3. Thus, it was considered that the grade of eye disorder did not influence recovery or improvement.

Although no skin malignancies were observed in this study, these were reported with a median onset of 407.5 days (range: 30–1905 days) in the COLUMBUS trial [11]. It is possible that the observation period of our study was not sufficient to detect such events.

The ORR was lower in this study compared with the COLUMBUS trial, possibly due to differences in patient characteristics. For example, in our study, 37.8% of patients had received third-line treatment or later, 8.1% of patients had an ECOG PS ≥ 2, and 14.5% of cases were not evaluable. OS outcomes were inconclusive due to a high number of unknown outcomes and the 12-month observation period limited our ability to compare our results with those of the COLUMBUS trial.

Several limitations of this study include the short 12-month observation period and the absence of a control group. Best tumour responses were only collected according to RECIST ver. 1.1 criteria and were not adjudicated centrally. The exact timing for evaluating effectiveness was not specified in the study plan and the 12-month follow-up period was considered limited for evaluating OS.

## Conclusions

The results of this PMS in real-world clinical practice in Japan did not reveal any additional safety concerns compared with clinical trials of the combination treatment of encorafenib plus binimetinib in patients with *BRAF*-mutant malignant melanoma. Although eye disorders often occurred early in the treatment, most cases recovered or improved with drug discontinuation, interruption, dose reduction or continuation.

## Supplementary Information

Below is the link to the electronic supplementary material.

Supplementary file1 (DOCX 244 KB)

Supplementary file2 (DOCX 25 KB)

## Data Availability

The authors confirm that the data supporting the findings of this study are available within the article.
