# Supplementary material for: Post-marketing surveillance of encorafenib in combination with binimetinib in Japanese patients with BRAF-mutant melanoma
Source: Int J Clin Oncol. 2025 Feb 7;30(4):814–23. doi: 10.1007/s10147-025-02693-6 (PMC11946937; doi:10.1007/s10147-025-02693-6)
Supplement: Supplementary file 1 — Supplementary file1 (DOCX 244 KB) [file 10147_2025_2693_MOESM1_ESM.docx]

**Supplementary Figure 1.** Eye Disorders by PT Specification of ≥1.5% - time to onset (N=172)

**
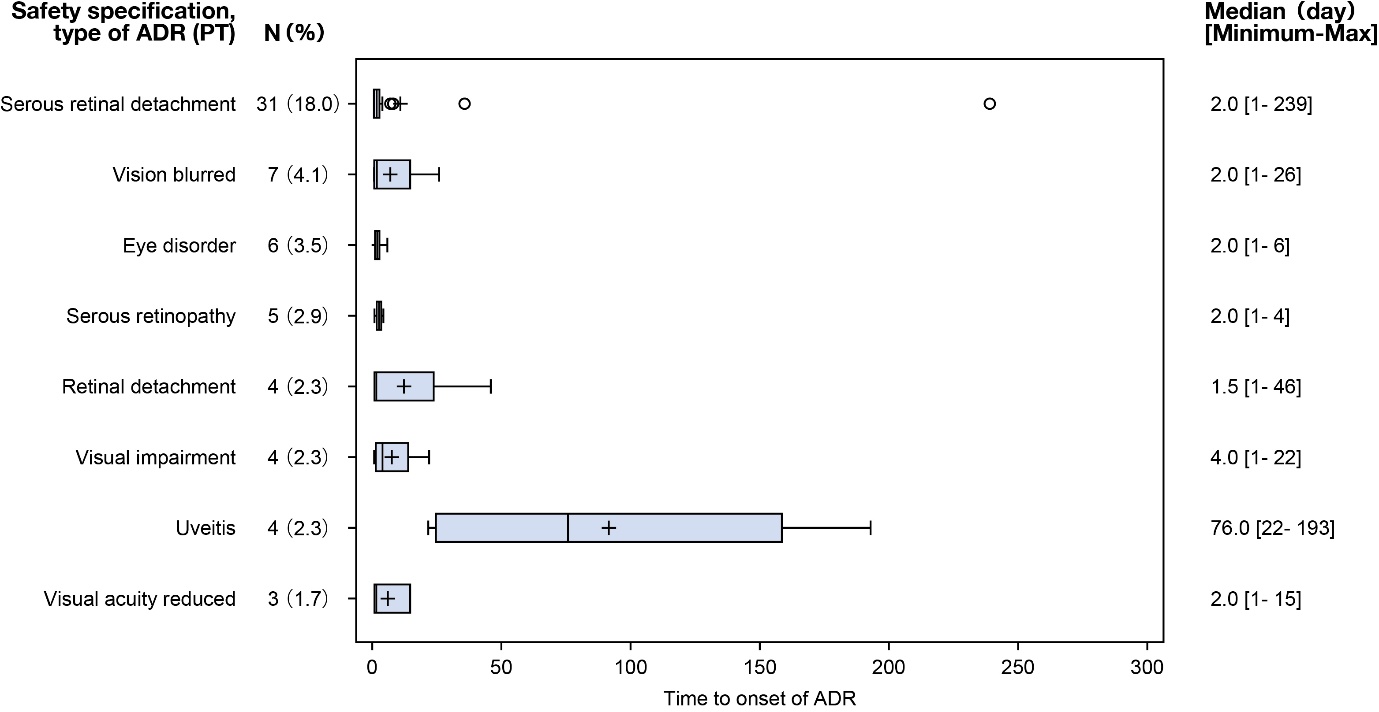
**

**Supplementary Figure 2.** Time to resolution or improvement of eye disorders by PT specifications (≥1.5%)


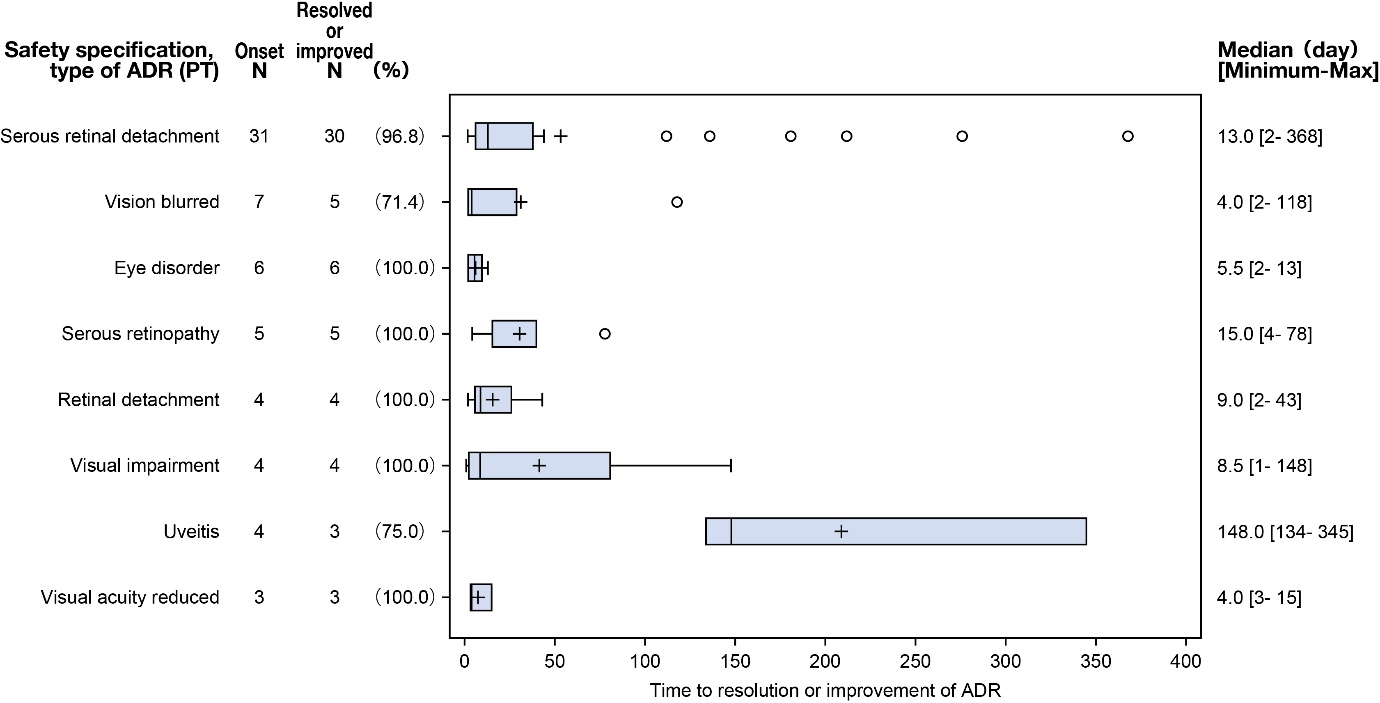


PT: Preferred term.
