# Supplementary material for: Post-marketing surveillance of encorafenib in combination with binimetinib in Japanese patients with BRAF-mutant melanoma
Source: Int J Clin Oncol. 2025 Feb 7;30(4):814–23. doi: 10.1007/s10147-025-02693-6 (PMC11946937; doi:10.1007/s10147-025-02693-6)
Supplement: Supplementary file 2 — Supplementary file2 (DOCX 25 KB) [file 10147_2025_2693_MOESM2_ESM.docx]

**Supplementary Table 1.** Definitions of ADRs included in the safety specifications

| Adverse drug reaction | Definition |
| --- | --- |
| Malignant skin tumours | Primary HLGT: Skin neoplasms malignant and unspecified |
| Palmar-plantar erythrodysaesthesia syndrome | PT: Palmar-plantar erythrodysaesthesia syndrome |
| Eye disorders | Primary SOC: Eye disorders |
| Cardiac dysfunction | SMQ: Cardiac failure (broad scope) |
| Hypertension | SMQ: Hypertension (narrow scope) |
| Rhabdomyolysis | SMQ: Rhabdomyolysis/myopathy (broad scope) |
| Hepatic dysfunction | SMQ: Liver related investigations, signs and symptoms (narrow scope)  SMQ: Cholestasis and jaundice of hepatic origin (narrow scope)  SMQ: Hepatitis, non-infectious (narrow scope)  SMQ: Hepatic failure, fibrosis and cirrhosis and other liver damage-related conditions (narrow scope) |
| Haemorrhage | SMQ: Haemorrhage terms (excluding laboratory terms) (narrow scope)” |

ADR, adverse drug reaction, HLGT: High Level Group Term, PT, Preferred term, SMQ: Standardised MedDRA Queries, SOC: System Organ Class.

**Supplementary Table 2.** Eye damage of ≥1.5% according to PT specification: outcome

| **Safety specification, type of ADR (PT)** | **Outcome** | | | | | | |
| --- | --- | --- | --- | --- | --- | --- | --- |
|  | **Total No. of patients** | **Resolved/Resolving** | **Not resolved** | **Resolved with sequalae** | | **Death due to the event** | **Unknown** |
| Eye disorders, *n* (%) |  |  |  |  | |  |  |
| Serous retinal detachment | 31 | 30 (96.8) |  | 1 (3.2) | |  |  |
| Vision blurred | 7 | 5 (71.4) | 1 (14.3) |  | |  | 1 (14.3) |
| Eye disorder | 6 | 6 (100) |  |  | |  |  |
| Serous retinopathy | 5 | 5 (100.0) |  |  | |  |  |
| Retinal detachment | 4 | 4 (100.0) |  |  | |  |  |
| Visual impairment | 4 | 4 (100.0) |  |  |  | |  |
| Uveitis | 4 | 3 (75.0) | 1 (25.0) |  |  | |  |
| Visual acuity reduced | 3 | 3 (100.0) |  |  |  | |  |

^a^ *n (%)*

ADR: adverse drug reaction, PT: Preferred term.

**Supplementary Table 3.** Outcomes of safety specification of eye disorders by grade

| **Safety Specification** | **Grade** | **Resolved or improved, *n* (%)** | | **Time to resolution / improvement, median [min−max]** |
| --- | --- | --- | --- | --- |
| Eye disorders | 1 | 36 | 97.3% | 12.0 [1–368] |
|  | 2 | 19 | 95.0% | 10.0 [2–136] |
|  | 3 | 11 | 91.7% | 15.0 [1–345] |
|  | 4 | 1 | 100.0% | 43.0 [43– 43] |

**Supplementary Table 4.** Safety specification: status at the time of onset of eye disorders, treatment for eye disorders, PT any grade incidence ≥1.5%

| Adverse drug reaction | Resolution and improvement rates by treatment status | | | | | | | |
| --- | --- | --- | --- | --- | --- | --- | --- | --- |
|  | Continued, etc. | | Dose reduction | | interruption | | Discontinuation | |
|  | Treatment | Resolved / improved, *n* (%) | Treatment | Resolved / improved, *n* (%) | Treatment | Resolved / improved, *n* (%) | Treatment | Resolved / improved, *n* (%) |
| Eye disorder | 33 | 32 （97.0） | 9 | 9 (100） | 35 | 34 （97.1） | 19 | 16 （84.2） |
| Serous retinal detachment | 12 | 12 （100） | 5 | 5 （100） | 17 | 17 （100） | 8 | 6 （75.0） |
| Vision blurred | 4 | 3 （75.0） | - | - | 3 | 2 （66.7） | - | - |
| Eye disorder | 3 | 3 （100） | 2 | 2 （100） | 2 | 2 （100） | - | - |
| Serous retinopathy | 3 | 3 （100） | - | - | 1 | 1 （100） | 1 | 1 （100） |
| Retinal detachment | 3 | 3 （100） | - | - | 1 | 1 （100） | 1 | 1 （100） |
| Uveitis | 1 | 1 （100） | - | - | 1 | 1 （100） | 2 | 1 （50.0） |
| Visual impairment | 1 | 1 （100） | - | - | 3 | 3 （100） | - | - |
| Visual acuity reduced | 3 | 3 （100） | - | - | - | - | - | - |

PT: Preferred term.

**Supplementary Table 5.** Safety specification: onset status of eye complications and eye disorders, PT any grade, incidence ≥1.5%

|  |  | Eye complications | | |
| --- | --- | --- | --- | --- |
| Adverse drug reaction | Any grade, *n* (%) | Absent (n=156) | Present (n=15) | Unkown (n=1) ^a^ |
| Serous retinal detachment | 31（18.0） | 30（19.2） | 1（6.7） | - |
| Vision blurred | 7（4.1） | 5（3.2） | 2（13.3） | - |
| Eye disorder | 6（3.5） | 6（3.8） | - | - |
| Serous retinopathy | 5（2.9） | 5（3.2） | - | - |
| Retinal detachment | 4（2.3） | 4（2.6） | - | - |
| Uveitis | 4（2.3） | 3（1.9） | 1（6.7） | - |
| Visual impairment | 4（2.3） | 4（2.6） | - | - |
| Visual acuity reduced | 3（1.7） | 2（1.3） | 1（6.7） | - |

^a^No eye disorders were observed.

PT: Preferred term.
